# Microbial communities in dark oligotrophic volcanic ice cave ecosystems of Mt. Erebus, Antarctica

**DOI:** 10.3389/fmicb.2015.00179

**Published:** 2015-03-11

**Authors:** Bradley M. Tebo, Richard E. Davis, Roberto P. Anitori, Laurie B. Connell, Peter Schiffman, Hubert Staudigel

**Affiliations:** ^1^Division of Environmental and Biomolecular Systems, Institute of Environmental Health, Oregon Health & Science UniversityPortland, OR, USA; ^2^School of Marine Sciences, University of MaineOrono, ME, USA; ^3^Department of Geology, University of California, DavisDavis, CA, USA; ^4^Institute of Geophysics and Planetary Physics, Scripps Institution of OceanographyLa Jolla, CA, USA

**Keywords:** ribulose-1,5-bisphosphate carboxylase/oxygenase, RubisCO, carbon monoxide, oligotrophy, chemolithoautotrophy

## Abstract

The Earth's crust hosts a subsurface, dark, and oligotrophic biosphere that is poorly understood in terms of the energy supporting its biomass production and impact on food webs at the Earth's surface. Dark oligotrophic volcanic ecosystems (DOVEs) are good environments for investigations of life in the absence of sunlight as they are poor in organics, rich in chemical reactants and well known for chemical exchange with Earth's surface systems. Ice caves near the summit of Mt. Erebus (Antarctica) offer DOVEs in a polar alpine environment that is starved in organics and with oxygenated hydrothermal circulation in highly reducing host rock. We surveyed the microbial communities using PCR, cloning, sequencing and analysis of the small subunit (16S) ribosomal and Ribulose-1,5-bisphosphate Carboxylase/Oxygenase (RubisCO) genes in sediment samples from three different caves, two that are completely dark and one that receives snow-filtered sunlight seasonally. The microbial communities in all three caves are composed primarily of Bacteria and fungi; Archaea were not detected. The bacterial communities from these ice caves display low phylogenetic diversity, but with a remarkable diversity of RubisCO genes including new deeply branching Form I clades, implicating the Calvin-Benson-Bassham (CBB) cycle as a pathway of CO_2_ fixation. The microbial communities in one of the dark caves, Warren Cave, which has a remarkably low phylogenetic diversity, were analyzed in more detail to gain a possible perspective on the energetic basis of the microbial ecosystem in the cave. Atmospheric carbon (CO_2_ and CO), including from volcanic emissions, likely supplies carbon and/or some of the energy requirements of chemoautotrophic microbial communities in Warren Cave and probably other Mt. Erebus ice caves. Our work casts a first glimpse at Mt. Erebus ice caves as natural laboratories for exploring carbon, energy and nutrient sources in the subsurface biosphere and the nutritional limits on life.

## Introduction

Over the past decade, much evidence has accumulated that the Earth's crust hosts a deep biosphere with a substantial total biomass in sedimentary, volcanic and other crustal geological settings on continents or in the oceanic crust (Stevens and McKinley, 1995; Whitman et al., 1998; D'Hondt et al., 2009; Jørgensen, 2012; Kallmeyer et al., 2012). However our understanding of this dark biosphere is quite limited. How much biomass is produced from energy derived from the inorganic local environment rather than from introduced or photosynthetically-derived organic matter? Does this biomass have any impact on surface food webs? Volcanic settings, in particular, including the oceanic crust that comprises two-thirds of the Earth's surface, have attracted much attention, as they are extremely widespread. Volcanic rocks commonly host vigorously circulating hydrothermal systems and prolific aquifers, and are chemically more reactive than most other geologic systems, providing electron acceptors and donors for chemolithoautotrophic microbial communities thriving in the absence of light and hence photosynthetic primary production. It is not surprising that microbial fossils in volcanic rocks suggest that microbial activity dominates the alteration of volcanic glass and other reactive phases, a process that appears to have been active since the earliest periods of life documented on Earth (Furnes and Staudigel, 1999; Staudigel et al., 2008). Indeed volcanic systems have been the subject of key deep biosphere studies including Subsurface Lithoautotrophic Microbial Ecosystems (“SLiMEs”) (Stevens and McKinley, 1995).

Studies of life in the absence of photosynthetic primary production have focused largely on deep-sea ecosystems such as hydrothermal vents at spreading centers and seamounts or on subsurface terrestrial environments. Terrestrial caves, in particular, have attracted interest as a means to access and study the crustal biosphere and have been suggested to offer an analog to extraterrestrial subsurface life on planets such as Mars (Boston et al., 2001). Most cave environments are influenced by human activities and are either relatively shallow and/or at risk for introduction of organic matter from the Earth's surface, either from animals (e.g., bats) or from groundwater circulation. Although most often research has been done on caves with acidic or sulfidic conditions (Sarbu et al., 1996; Chen et al., 2009; Engel et al., 2009; Jones et al., 2012), a fewer oligotrophic environments such as carbonate caves have also been examined (Barton et al., 2004; Ortiz et al., 2014).

**Figure 1:**
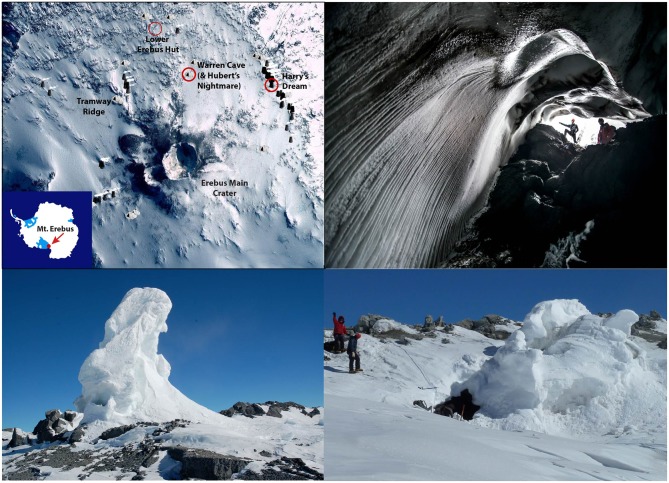
**A map of Mt. Erebus, Antarctica and pictures of the ice caves**. **Left top**: The locations of Mt. Erebus (inset) and of Warren Cave, Hubert's Nightmare, and Harry's Dream in relation to landmarks including the Lower Erebus Hut, the Erebus main crater and Tramway Ridge. Different cave sites on the map are indicated by triangles and ice towers are indicated by trapezoids. The base map is from http://erebuscaves.nmt.edu/. **Right top**: Pictures of one of the main entrances to Warren Cave (photo courtesy of Alasdair Turner). **Lower left**: Harry Dream. Note the small cave entrance at the lower left base of the ice chimney. **Lower right**: Hubert's Nightmare. The entrance to the cave is in the middle of the image.

The fumarolic ice caves on Mt. Erebus (Ross Island, Antarctica; Figure 1) are another example of an oligotrophic cave system, and are characterized by having volcanic rock and their weathered sediments as substrates. The Mt. Erebus ice caves are at high altitude in one of the most remote and oligotrophic environments on Earth and represent an excellent accessible model system for understanding fundamental microbe-mineral interactions contributing to the subsurface biosphere. This environment ensures that they are highly oligotrophic with almost no potential for the introduction of photosynthesis-based organic matter from invading animals or wash-down of organics from overlying soils. Mt. Erebus ice caves are moist, relatively warm habitats (on average ~0°C, Curtis and Kyle, 2011) that persist over decades (Lyon and Giggenbach, 1973) even though they are dynamic systems with cycles including collapse and post-collapse re-building. Sub-glacial fumaroles issue air-dominated gasses with 80–100% humidity and up to 3% CO_2_ (Curtis and Kyle, 2011; Curtis et al., 2013). The volcano source gas emissions, some of which may be entrained in the fumaroles, contain CO and H_2_, but are essentially devoid of CH_4_ and H_2_S (Oppenheimer and Kyle, 2008; Moussalam et al., 2012). Many of the caves are completely dark and therefore unable to support photosynthesis. In these DOVEs the only possible sources of organic carbon are from atmospheric deposition or ice algae that may grow on the surface of the ice during summer and subsequently be introduced into the caves through burial from above and melting from below. Thus, Mt. Erebus DOVEs provide an ideal ecosystem to study chemolithoautotrophic microorganisms that, in other cave and basaltic environments, would be masked by heterotrophic and photosynthetic organism biomass. Consequently they may shed new insights into the role of volcanoes and volcanic emissions in supporting life.

We surveyed the microbial communities in weathered sediments from 3 cave systems (Figure 1): (1) Harry's Dream, a shallow cave under the influence of indirect sunlight during the Antarctic summer; (2) Warren Cave, a completely dark cave studied extensively for its CO_2_ emissions and temperature fluctuations by the Mount Erebus Volcano Observatory (MEVO) (Curtis and Kyle, 2011; http://erebuscaves.nmt.edu/); and (3) Hubert's Nightmare, a small unmapped dark cave about 50 m west of Warren Cave. Samples from these cave environments were used to investigate the phylogenetic diversity and the primary carbon fixation pathways of the microbial communities to evaluate the possibility for chemolithoautotrophy. A recent report has described the presence of a moderate diversity of fungi in Warren Cave (Connell and Staudigel, 2013). This manuscript reports on the first identification of the prokaryotic communities in these cave environments.

## Materials and methods

### Sites and samples

Fumarolic ice caves and ice towers on Mt. Erebus are the result of a complex interaction between volcanic heat and outgassing, the volcano's snow and ice cover, and the extremely cold surrounding atmosphere. Volcanic heat and gas exhalations melt overlying ice and snow that produce liquid water that can percolate down into the volcano where it may encounter hot rocks that can turn it into steam. This steam mixes with volcanic gasses escaping from a magma chamber and the air contained in the porous rock. These warm, water- and CO_2_-rich gasses rise to the surface of the volcano, where they may melt a cave into the volcano's ice cover or directly escape into the atmosphere. Focused steam-rich vents located on rock exposure rapidly chill upon venting and will rapidly form an ice tower, effectively a chimney serving for the steam to escape. Temperatures in large ice caves remain relatively constant with major temperature drops only during major barometric pressure changes (Curtis and Kyle, 2011) suggesting that the feeder systems for these caves are largely isolated from the atmosphere. These caves are commonly within ice or snow packs ranging in thickness from meters to tens of meters known to persist over decades (Lyon and Giggenbach, 1973).

Sediment samples were collected during November 2010 from the floors of three ice caves on Mt. Erebus, Antarctica: Harrys Dream, Warren Cave and Hubert's Nightmare (Figure 1). Samples were also collected in November 2012 from 10 caves: Harry's Dream, Warren Cave, Kachina Cave, Worm Tongue, Haggis Hole, Helo Cave, Mammoth Cave, Hut Cave, Heroine Cave and Sauna Cave. In Harrys Dream, Warren Cave and Hubert's Nightmare the samples were collected from thermally active sediments as evidenced by temperature and/or emission of steam. Warren Cave was chosen for more detailed analysis as a large completely dark and well-studied cave with respect to CO_2_ emissions and temperature fluctuations by the MEVO (http://erebuscaves.nmt.edu/) (Curtis and Kyle, 2011).

### SSU rRNA gene methods

Environmental DNA was extracted from the sediments using the fastDNA Spin kit for sediments (Q-biogene, Carlsbad, CA). PCR reactions for amplification of community bacterial SSU rRNA genes contained environmental cave DNA, 1 X PCR buffer, 3 mM MgCl_2_, 250 μM of each dNTP, 10 μM of each primer (27f and 1492r; Table 1), and 0.5 U of *Taq* polymerase (DYnazyme II, Thermo Fisher Scientific, Lafayette, CO). Cycling conditions were a 94°C hot start for 2 min, followed by 25 cycles of 94°C for 30 s, 50°C for 30 s and 72°C for 2 min, and completed with a 5 min 72°C extension. For construction of SSU rRNA gene libraries for each cave sample, PCR products from multiple (4–8) clean PCR reactions (i.e., showing only a band of the expected size) were pooled, and purified using a PCR purification kit (QIAGEN, Valencia, CA). Libraries were generated using a TOPO TA cloning kit and chemically competent *Escherichia coli* TOP10F cells (Invitrogen, Carlsbad, CA). Cloned SSU rRNA genes were sequenced from both directions using primers T3 and T7, and the data used to assign OTUs using DOTUR (Schloss and Handelsman, 2005); a 97% sequence identity was used for OTU classification. One to three representative clones were chosen for each OTU and bidirectional sequencing of the complete gene performed (primers 704F and 926R, Table 1). Taxonomic assignments were determined to the phylum level using the SILVA search and classify function (v1.2.11) with a minimum identity value of 0.80 using the SILVA taxonomy for classification (Pruesse et al., 2012). A maximum likelihood tree was then calculated using RAxML v7.2.6 (Stamatakis, 2006) using the general time reversible model of nucleotide substitution (Tavaré, 1986) for OTUs representing more than two clones. The tree was calculated 10 consecutive times using randomized starting trees to find the tree with the greatest likelihood to be the most representative. The tree files were then visualized within the software Mega and annotated using the vector-drawing program Inkscape.

**Table 1:** **Primers used in this study**.

**Target**	**Primers**	**Sequence**	**References**
RubisCO *cbbL*	R1P1F	CARCCNTTCMWRCGBTGG	This Study
	R1P1R	GTNCCDCCDCCRAAYTG	This Study
RubisCO *cbbL*	R2P2F	AAGGAYGACGAGAACATCAAYT	This Study
	R2P2R	AAYCGSRTNGCSCTSGA	This Study
RubisCO *cbbM*	R3P1F	TTNTCRAAGAARCCNGGNA	This Study
	R3P1R	GGNACNATCATCAARCCNAA	This Study
Bacteria/universal SSU rRNA gene	27F	AGRGTTTGATCMTGGCTCAG	Modified from Lane (1991)
	Univ533F	GTGYCAGCMGCCGCGGTAA	Modified from Schmidt et al. (1991)
	1492R	RGYTACCTTGTTACGACTT	(Lane, 1991)
	Bact684R	TCTACGSATTTYACYSCTAC	Modified from Amann et al. (1990)
	704F	GTAGSRGTRAAATSCGTAGA	Modified from Lane (1991)
	926R	CCGYCWATTCMTTTRAGTTT	Modified from Lane (1991)
ATP Citrate Lyase *aclB*	178F	CCNGAYATGYTNTTTGGWAA	This Study
	1195R	CCNWNYTCRTARTTWGGNCC	This Study
Archaea SSU rRNA gene	21F	TTCYGGTTGATCCYGCCRGA	(Delong, 1992)
	922R	YCCGGCGTTGANTCCAATT	(Moyer et al., 1998)

### Q-PCR methods

Bacterial biomass was estimated using a method modified from Einen et al. (2008) where ribosomal RNA genes are quantified using quantitative PCR (Q-PCR) and extrapolated to cells/g of material. Q-PCR reactions were performed using a StepOnePlus real-time PCR system (Life Technologies) with primers Univ533F and Bact684R (Table 1), DyNAmo Flash SYBR green PCR master mix (ThermoFisher Scientific, Waltham, MA) with absolute quantification against a standard curve. Each reaction contained 1X master mix, 0.5 μM of each primer, 1X ROX reference dye, and 1ng of environmental cave DNA or a serial dilution of plasmid DNA. Q-PCR reactions were 95° for 7 min as a hot start, followed by 35 cycles of 95°C for 5 s and 60°C for 30 s. PCR efficiency was 84%. Bacterial counts were then extrapolated to biomass using the conversion factor of 3.9 copies of rRNA genes/bacterial genome (Einen et al., 2008).

### RubisCO and *aclB*

PCR amplification was attempted for the two main types of autotrophic CO_2_ fixation mechanisms, RubisCO (Calvin-Benson-Bassham cycle) and ATP citrate lyase (reverse TCA cycle) to determine the possible presence of different carbon fixation pathways (Table 1). Each PCR reaction contained 1 U of Phire polymerase (Thermo Fisher Scientific, Waltham, MA), 1 X PCR Buffer, 200 μM dNTP, 2 μM each primer, 5 μg BSA, and 1 ng/μl environmental cave DNA. Cycling conditions began with a 2 min hot start at 98°C, followed by 30 cycles of 98°C for 10 s, 60°C for 20 s, and 72°C for 30 s and completed with a 2 min extension at 72°C. PCR product from positive reactions were excised from a 1.5% agarose gel and purified using the GeneJET extraction kit (Thermo Fisher Scientific). Purified PCR products were then ligated into the pJET1.2 vector (Thermo Fisher Scientific) at a 3:1 ratio and transformed into chemically competent cells (Active Motif, Carlsbad, CA). Both strands of inserts from 48 clones were sequenced from each clone library using primers Pjet1.2F and Pjet1.2R. The sequences were then translated and aligned with representative sequences from described isolates and uncultured clones. Phylogenetic trees were calculated using RaxML v7.0.3 (Stamatakis, 2006) using the Whelan and Goldman model of amino acid substitution (Whelan and Goldman, 2001).

### Nucleotide sequence accession numbers

The SSU rRNA and the Rubisco sequences representing the OTUs analyzed in this paper have been submitted to GenBank and assigned accession numbers KJ623626—KJ623652 and KJ623653—KJ623698, respectively.

### Chemical characterization

We analyzed glasses from grain mounts of all the caves visited in 2012 (except Hubert's Nightmare) with the Cameca SX-100 electron microprobe at University of California at Davis operated at 15 keV, 10 nA, and a spot size of 10 μm. Calibration standards included silicate minerals, natural basaltic glass, oxides, and sulfides. Counting times on peak and background ranged from 10 to 50 s. We analyzed two spots on three separate grains for each cave sample. Each value given in Table 2 represents a linear average of these six individual measurements. The standard deviation for all six measurements are typically equal or better than 0.3 for SiO_2_; 0.1 for Al_2_O_3_; 0.04 for TiO_2_; 0.03 for MgO; 0.04 for CaO, 0.05 for MnO; 0.1 for FeO, 0.17 for Na_2_O, 0.16 for K_2_O, 0.02 for P_2_O_5_, 0.008 for S and 0.012 for Cl. This variance is generally less for the duplicate measurements on a particular grain but may double in different grains for some elements from Worm Tongue and Haggis Hole, likely representing slight compositional variation between the grains chosen for analysis.

**Table 2:** **Sediment composition**.

**Cave^#^**	hv^*^	**S**	**Cl**	**Na_2_O**	**K_2_O**	**MgO**	**CaO**	**MnO**	**FeO**	**Al_2_O_3_**	**SiO_2_**	**TiO_2_**	**P_2_O_5_**	**Total**
Kachina	No	0.04	0.15	8.79	5.40	0.88	1.93	0.25	5.43	19.91	56.27	1.03	0.26	100.34
Worm Tongue	No	0.04	0.16	8.74	5.43	0.94	2.02	0.25	5.59	19.84	55.89	1.08	0.27	100.27
Haggis Hole	Yes	0.04	0.16	8.85	5.34	0.92	2.06	0.27	5.47	19.85	56.02	1.04	0.25	100.28
Harry's Dream	Yes	0.04	0.16	8.82	5.41	0.87	1.92	0.26	5.50	19.80	56.12	0.99	0.27	100.15
Helo	No	0.04	0.15	8.80	5.36	0.86	1.95	0.24	5.46	20.01	56.20	1.06	0.24	100.38
Mammoth	No	0.04	0.15	8.82	5.35	0.89	1.91	0.28	5.42	19.71	56.17	1.01	0.23	99.98
Hut	Yes	0.04	0.15	8.80	5.27	0.87	2.00	0.27	5.27	19.88	56.13	1.01	0.23	99.93
Heroine	Yes	0.04	0.15	8.78	5.34	0.94	2.10	0.27	5.38	19.84	55.75	1.07	0.28	99.93
Warren	No	0.04	0.15	8.69	5.42	0.87	1.93	0.28	5.43	19.82	56.09	1.02	0.26	100.01
Sauna	Yes	0.00	0.00	7.28	2.85	0.01	3.68	0.00	0.20	22.89	63.25	0.11	0.02	100.31
Average (except Sauna)	–	0.04	0.15	8.79	5.37	0.89	1.98	0.26	5.44	19.85	56.07	1.03	0.26	100.14

# *Cave locations are mapped on the Mount Erebus Volcano Observatory website (http://erebuscaves.nmt.edu/)*.

* *Denotes the presence or absence of light from where the soil was collected*.

## Results and discussion

### Cave characteristics

Our microbiological samples were extracted from sandy sediments taken from sub-ice fumarolic vents in the summit region of Mt Erebus. In order to contrast and compare sub-ice lava flow compositions with their subaerial counterparts in the partly ice-covered summit region, we analyzed materials from 10 ice caves (Table 2). Our analyses of phonolite glasses from nine caves display very little compositional variation (Table 2), all of them largely the same as the subaerially exposed lava flows analyzed by Kelly et al. (2008). Hence, microbial samples studied here from Warren Cave, Hubert's Nightmare and Harry's Dream effectively have the same substrates, a typical Mt Erebus anorthoclase phonolite, with only minor variability within and between caves. We note the relatively high concentrations of iron and manganese in the phonolitic glasses that can be expressed as the divalent FeO (5.4 wt%) and MnO (0.26 wt %) and which may serve as energy sources for microbial growth.

Of the 3 caves studied for microbiology, our sediment samples from Warren Cave registered the warmest temperature we recorded (Table 3). Warren Cave is also the largest of these caves and although the average air temperature in the cave is close to 0°C, the sampled sandy sediments are quite warm (14.6°C). The total organic carbon (TOC) content of the Warren Cave and Harry's Dream sediment samples were 126 and 78 μg/g respectively, firmly at the lower end of the 50–1690 μg/g levels reported for other volcanic sediments, e.g., Antarctica Taylor Valley soils (Burkins et al., 2001; Connell et al., 2006) and the Atacama desert (Lynch et al., 2012, and references cited therein). Although we only have detailed sediment analyses for Warren Cave and Harry's Dream, we anticipate the levels of total organic carbon to be similarly low in Hubert's Nightmare, given the remoteness of Mt. Erebus and the lower temperatures of the sediment.

**Table 3:** **Properties of the samples for microbiological analysis**.

**Cave**	**Coordinates (Elevation)**	**Temperature air#/sediment (°**C**)**	**pH**	**TOC (μg/g sediment)**	# of Bacteria^*^(x 10^6^/g sediment)
Warren Cave	S77°31.003, E167°09.884 (3470.5 m)	14.6/18.5	5.2	126	40.0
Harry's Dream	S77°31.016, E 167°13.087 (3458 m)	10.4/10.4	5.2	78	2.8
Hubert's Nightmare	S77°31.003, E167°09.884 (3470.5 m)	−0.8/0.1	5.9	ND	1.6

*^#^Air temperature above sediment sample*.

* *Archaea were not detected in any of the 3 caves*.

*ND, not determined; TOC, total organic carbon*.

### Bacterial abundance and microbial diversity based on SSU rRNA genes

**Figure 2:**
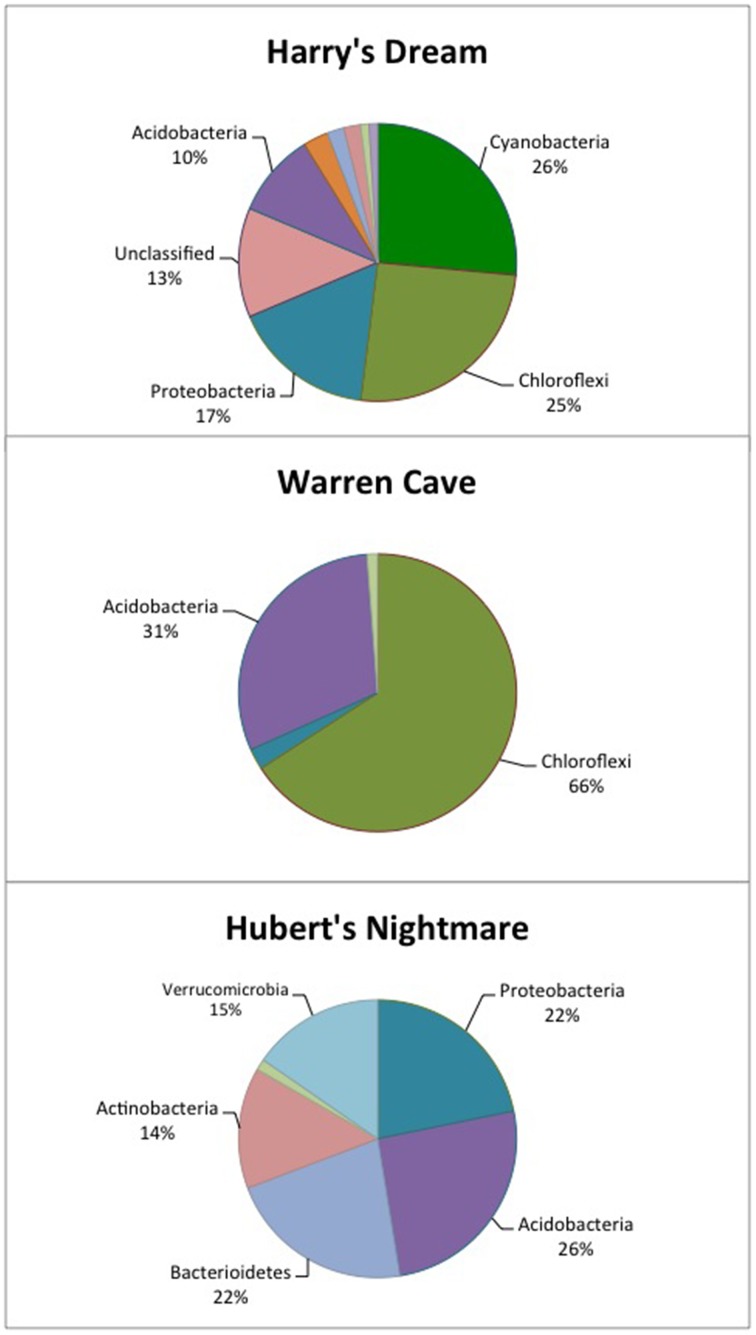
**Pie chart showing the percentage of the major phyla identified in each cave by 16S rDNA analysis**. The identification of the other, minor phyla are given in Tables 4–6.

**Figure 3:**
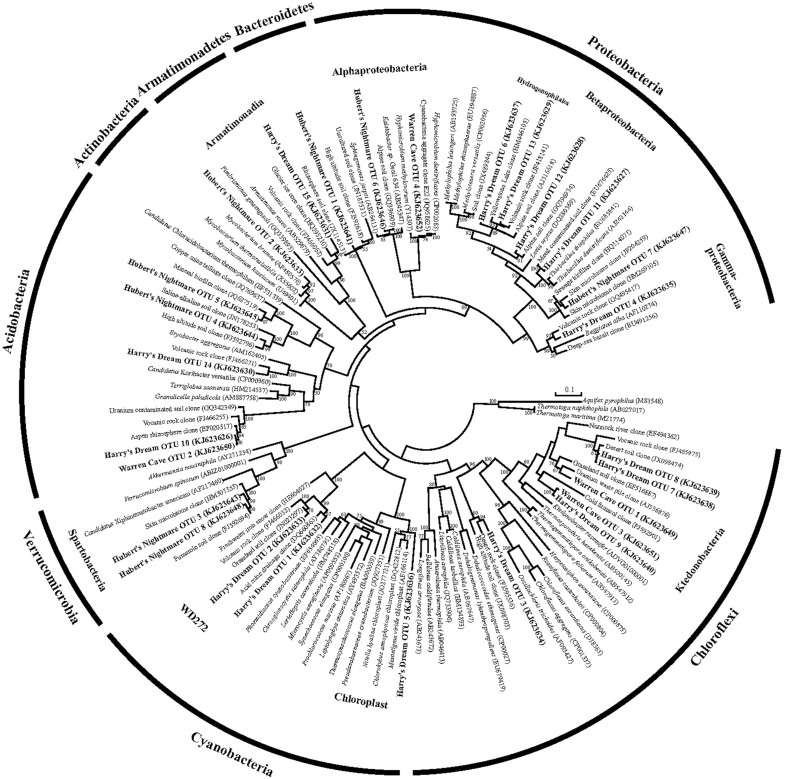
**Phylogenetic tree of the small subunit SSU ribosomal RNA genes of Bacteria from the caves**.

We used molecular phylogenetic analysis to examine the microbial communities in the three caves. The bacterial small subunit (SSU) ribosomal RNA gene was PCR amplified using domain specific primers (Table 1). PCR amplifications using Archaea-specific primers were negative for all three caves. Bacterial numbers for the cave samples determined by quantitative PCR (Q-PCR) of small subunit ribosomal genes (Einen et al., 2008) were 1.6–40 × 10^6^ cells/g sediment (Table 3), or up to about 10 fold higher than found in the glassy rind of seafloor basalts (Einen et al., 2008). The PCR products were cloned and sequenced (results for the Eukarya are reported elsewhere, Connell and Staudigel, 2013). For Harry's Dream, Warren Cave, and Hubert's Nightmare we sequenced 102, 82, and 78 clones which grouped into, respectively, 34, 11, and 18 Operational Taxonomic Units (OTUs; 97% sequence identity cutoff) (Tables 4–6) and Chao1 indices of 70.1, 35.5, and 58.5. Distinct Bacterial communities were identified in each cave (Figure 2). The phylogeny of the organisms found in the clone libraries was frequently similar to cultured organisms or 16S rRNA gene sequences from other volcanic, cave, sediment or cold environments (Figure 3).

**Table 4:** **Harry's Dream clone identification**.

**OTU ID**	**# Clones**	**% Library**	**RepClone**	**SilvaClass**
HD1	19	18.6	HDclone4	Bacteria;*Cyanobacteria*;WD272;
HD2	13	12.7	HDclone9	Bacteria;
HD3	9	8.8	HDclone2	Bacteria;*Chloroflexi*;
HD4	6	5.9	HDclone3	Bacteria;*Proteobacteria*;Gammaproteobacteria;
HD5	6	5.9	HDclone5	Bacteria;*Cyanobacteria*;Chloroplast;
HD6	5	4.9	HDclone12	Bacteria;*Proteobacteria*;*Betaproteobacteria*;Hydrogenophilales;Hydrogenophilaceae;
HD7	5	4.9	HDclone19	Bacteria;*Chloroflexi*;*Ktedonobacteria*;Ktedonobacterales;
HD8	4	3.9	HDclone10	Bacteria;*Chloroflexi*;*Ktedonobacteria*;Ktedonobacterales;
HD9	3	2.9	HDclone43	Bacteria;*Chloroflexi*;*Ktedonobacteria*;Ktedonobacterales;
HD10	3	2.9	HDclone29	Bacteria;*Acidobacteria*;*Acidobacteria*;subgroup 2;
HD11	2	2.0	HDclone141	Bacteria;*Proteobacteria*;*Betaproteobacteria*;Hydrogenophilales;Hydrogenophilaceae; Thiobacillus;
HD12	2	2.0	HDclone53	Bacteria;*Proteobacteria*;*Betaproteobacteria*;
HD13	2	2.0	HDclone13	Bacteria;*Chloroflexi*;*Ktedonobacteria*;Ktedonobacterales;
HD14	2	2.0	HDclone37	Bacteria;*Acidobacteria*;*Acidobacteria*;subgroup 3;Family Incertae Sedis;Candidatus Solibacter;
HD15	2	2.0	HDclone55	Bacteria;Armatimonadetes;Armatimonadia;Armatimonadales;
HD16	1	1.0	HDclone18	Bacteria;SM2F11;
HD17	1	1.0	HDclone146	Bacteria;*Bacteroidetes*;Sphingobacteriia;Sphingobacteriales;Chitinophagaceae; Flavisolibacter;
HD18	1	1.0	HDclone150	Bacteria;*Bacteroidetes*;Sphingobacteriia;Sphingobacteriales;Chitinophagaceae; uncultured;
HD19	1	1.0	HDclone142	Bacteria;Planctomycetes;Planctomycetacia;Planctomycetales;Planctomycetaceae;
HD20	1	1.0	HDclone24	Bacteria;*Acidobacteria*;*Acidobacteria*;subgroup 4;RB41;
HD21	1	1.0	HDclone144	Bacteria;Armatimonadetes;
HD22	1	1.0	HDclone185	Bacteria;*Proteobacteria*;*Alphaproteobacteria*;Rhodospirillales;Acetobacteraceae;
HD23	1	1.0	HDclone158	Bacteria;*Proteobacteria*;*Betaproteobacteria*;Burkholderiales;
HD24	1	1.0	HDclone166	Bacteria;*Chloroflexi*;
HD25	1	1.0	HDclone7	Bacteria;*Chloroflexi*;*Ktedonobacteria*;Ktedonobacterales;
HD26	1	1.0	HDclone65	Bacteria;*Actinobacteria*;Thermoleophilia;Solirubrobacterales;
HD27	1	1.0	HDclone30	Bacteria;*Actinobacteria*;Acidimicrobiia;Acidimicrobiales;uncultured;
HD28	1	1.0	HDclone168	Bacteria;*Chloroflexi*;JG37-AG-4;
HD29	1	1.0	HDclone160	Bacteria;*Cyanobacteria*;WD272;
HD30	1	1.0	HDclone163	Bacteria;*Cyanobacteria*;WD272;
HD31	1	1.0	HDclone14	Bacteria;*Acidobacteria*;*Acidobacteria*;subgroup 2;
HD32	1	1.0	HDclone171	Bacteria;*Acidobacteria*;*Acidobacteria*;*Acidobacteria*les;*Acidobacteria*ceae (subgroup 1);Acidobacterium;
HD33	1	1.0	HDclone6	Bacteria;*Acidobacteria*;*Acidobacteria*;*Acidobacteria*les;*Acidobacteria*ceae (subgroup 1);Granulicella;
HD34	1	1.0	HDclone31	Bacteria;*Acidobacteria*;*Acidobacteria*;*Acidobacteria*les;*Acidobacteria*ceae (subgroup 1);Granulicella;

**Table 5:** **Warren Cave clone identification**.

**OTUID**	**# Clones**	**% Library**	**Repclone**	**SilvaClass**
WC1	33	40.2	WCclone11	Bacteria;*Chloroflexi*;*Ktedonobacteria*;Ktedonobacterales;
WC2	21	25.6	WCclone9	Bacteria;*Acidobacteria*;*Acidobacteria*;subgroup 2;
WC3	19	23.2	WCclone6	Bacteria;*Chloroflexi*;*Ktedonobacteria*;Ktedonobacterales;
WC4	2	2.4	WCclone43	Bacteria;*Proteobacteria*;*Alphaproteobacteria*;Rhizobiales;Hyphomicrobiaceae; yphomicrobium;
WC5	1	1.2	WCclone149	Bacteria;*Chloroflexi*;JG37-AG-4;
WC6	1	1.2	WCclone145	Bacteria;*Chloroflexi*;JG37-AG-4;
WC7	1	1.2	WCclone158	Bacteria;*Acidobacteria*;*Acidobacteria*;subgroup 6;
WC8	1	1.2	WCclone90	Bacteria;*Acidobacteria*;*Acidobacteria*;subgroup 3;Family Incertae Sedis;Candidatus Solibacter;
WC9	1	1.2	WCclone39	Bacteria;*Acidobacteria*;*Acidobacteria*;*Acidobacteria*les;*Acidobacteria*ceae (subgroup 1);uncultured;
WC10	1	1.2	WCclone148	Bacteria;Planctomycetes;BD7-11;
WC11	1	1.2	WCclone95	Bacteria;*Acidobacteria*;*Acidobacteria*;subgroup 2;

**Table 6:** **Hubert's Nightmare clone identification**.

**OTUID**	**# Clones**	**% Library**	**RepClone**	**SilvaClass**
HN1	16	20.3	HNclone2	Bacteria;*Bacteroidetes*;Sphingobacteriia;Sphingobacteriales;Chitinophagaceae;uncultured;
HN2	10	12.7	HNclone20	Bacteria;*Actinobacteria*;*Actinobacteria*;Corynebacteriales;Mycobacteriaceae; Mycobacterium;
HN3	9	11.4	HNclone13	Bacteria;*Verrucomicrobia*;Spartobacteria;Chthoniobacterales;DA101 soil group;
HN4	9	11.4	HNclone1	Bacteria;*Acidobacteria*;*Acidobacteria*;subgroup 4;RB41;
HN5	9	11.4	HNclone29	Bacteria;*Acidobacteria*;*Acidobacteria*;subgroup 4;Family Incertae Sedis;Blastocatella;
HN6	8	10.1	HNclone5	Bacteria;*Proteobacteria*;*Alphaproteobacteria*;Sphingomonadales;Sphingomonadaceae; Sphingomonas;
HN7	5	6.3	HNclone10	Bacteria;*Proteobacteria*;*Betaproteobacteria*;SC-I-84;
HN8	3	3.8	HNclone53	Bacteria;*Verrucomicrobia*;Spartobacteria;Chthoniobacterales;DA101 soil group;
HN9	1	1.3	HNclone137	Bacteria;*Acidobacteria*;*Acidobacteria*;subgroup 4;Family Incertae Sedis;Blastocatella;
HN10	1	1.3	HNclone35	Bacteria;Planctomycetes;Phycisphaerae;WD2101 soil group;
HN11	1	1.3	HNclone134	Bacteria;*Proteobacteria*;*Alphaproteobacteria*;Rhizobiales;
HN12	1	1.3	HNclone126	Bacteria;*Acidobacteria*;Holophagae;subgroup 7;
HN13	1	1.3	HNclone125	Bacteria;*Actinobacteria*;Thermoleophilia;Solirubrobacterales;
HN15	1	1.3	HNclone138	Bacteria;*Proteobacteria*;*Betaproteobacteria*;Methylophilales;Methylophilaceae;
HN16	1	1.3	HNclone26	Bacteria;*Proteobacteria*;*Betaproteobacteria*;Burkholderiales;
HN17	1	1.3	HNclone64	Bacteria;*Proteobacteria*;*Betaproteobacteria*;SC-I-84;
HN18	1	1.3	HNclone129	Bacteria;*Bacteroidetes*;Sphingobacteriia;Sphingobacteriales;Chitinophagaceae;Flavitalea;

The highest diversity of clones was found in Harry's Dream (Figure 2, Table 4). This finding is consistent with Harry's Dream being a more energy (light)-rich environment and, in fact, it was the only cave where representatives of well-recognized phototrophs (*Cyanobacteria*) were abundant. *Chloroflexi* were also abundant in this cave, but while there are known phototrophic members in this phylum they are anoxygenic phototrophs which would not be expected to be present. Rather, the vast phylogenetic diversity in the phylum *Chloroflexi* suggests broad metabolic capabilities within this group. The Harry's Dream *Chloroflexi* were dominated by the *Ktedonobacteria* class of Bacteria, representing about 58% of the *Chloroflexi* sequences (Table 4). Harry's Dream also had the only group of unclassified Bacteria for any of the three caves. The other abundant phyla include the *Proteobacteria* (primarily *Betaproteobacteria*) and the *Acidobacteria*.

In Warren Cave, the majority of the sequences fell into two phyla, the *Chloroflexi* (almost entirely *Ktedonobacteria*) and the *Acidobacteria*, representing about 66% and 31% of all the sequences, respectively. Only two minor OTUs were from other phyla: OTU4 was closely related to various *Hyphomicrobium* spp. (*Alphaproteobacteria*), a taxon known to oxidize manganese (Ghiorse and Ehrlich, 1992), while OTU10 (*Planctomycetes*) displayed no cultured relatives. *Chloroflexi* 16S rRNA gene sequences are often found in environmental DNA surveys and metagenomes (Huber et al., 2006), even in Antarctica (Cary et al., 2010; Pearce et al., 2012), but are usually only a minor part of the microbial community, so finding them in such relative abundance here was surprising. *Acidobacteria* are often the most abundant bacterial phylum in soils (Janssen, 2006) and are generally thought to be versatile aerobic heterotrophs adapted to low-nutrient conditions (Ward et al., 2009). They were the only group present in all three caves. Both the *Chloroflexi* and *Acidobacteria* phyla contain a large diversity of uncultured representatives, so the scope of the metabolic diversity within these phyla remains unknown, but their abundance implies that the Warren Cave environment provides conditions for their success.

Interestingly, in Hubert's Nightmare, a dark, but much colder cave with significantly lower airflow, the microbial community was quite distinct from Warren Cave even though they are adjacent to (within about 50 m of) each other. There were several phyla that were present or in much greater abundance than the other caves: *Verrucomicrobia, Actinobacteria*, and *Bacteroidetes*. There was also an abundance of *Proteobacteria* clones in this sample that included roughly equal percentages of *Betaproteobacteria* and *Alphaproteobacteria*; Hubert's Nightmare was the only cave with more than two clones from the *Alphaproteobacteria*. Whether the difference in the microbial community in Hubert's Nightmare as compared to Warren Cave was due to its shallower sediment depth, its lower degree of weathering (the sediment was coarser), the colder temperature or some other environmental factor can only be speculated upon.

### Screening for potential chemolithoautotrophy based on carbon fixation genes

**Figure 4:**
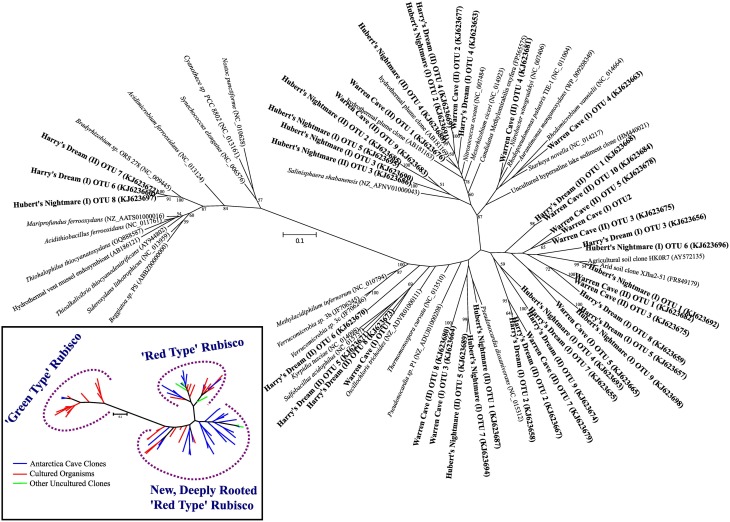
**Phylogenetic tree of RubisCO genes showing the sequences from the three caves in relation to previously known sequences**. Inset: a conceptualized version of the phylogenetic tree of the RubisCO genes showing the main branches of RubisCO gene sequences from cultured organisms (red lines), previously identified uncultured environmental sequences (green lines) and novel sequences from the Mt. Erebus caves (blue lines).

We screened for the presence of potential chemolithoautotrophic microorganisms in the three caves using PCR amplification of key genes for the dominant autotrophic CO_2_ fixing pathways: Those encoding ribulose-1,5-bisphosphate carboxylase/oxygenase (RubisCO) form I and form II (*cbbL* and *cbbM*, respectively), the primary CO_2_ fixing enzyme found in aerobic chemoautotrophs, and for ATP citrate lyase (*aclB*) diagnostic of the reverse tricarboxylic acid (rTCA) cycle, another key pathway important in CO_2_ fixation in both aerobes and anaerobes. RubisCO *cbbL* genes were detected, while PCR amplification of *aclB* was negative in all samples. Among the known types of RubisCO there are two major forms that catalyze net CO_2_ fixation (Tabita et al., 2008). Form I, comprised of 8 large and 8 small subunits, is typically found in aerobic bacteria and chloroplasts and is believed to be the more recently evolved aerotolerant form with a higher specificity for carboxylase activity than for oxygenase activity. Among the Form I RubisCOs are the “red” and “green” types. The green type Form I RubisCO is the common RubisCO in plants, green algae, cyanobacteria and some purple bacteria while the red type Form I RubisCO is found in non-green algae and includes some autotrophic *Alpha*- and *Beta-proteobacteria*. Form II, comprised of large subunit dimers, is most frequently found in organisms living in environments with relatively high CO_2_ concentrations, including anoxic and low oxygen environments and is generally believed to be the more ancient form. Three additional forms of RubisCO, forms III, IV and a hybrid form II/III, whose role in CO_2_ fixation remains unclear have been described (Tabita et al., 2008; Wu et al., 2009). Most of the RubisCO sequences found were related to the form I red type RubisCO (Figure 4). The majority of the Form I green type RubisCO were from Harry's Dream, which is not surprising given that it is seasonally influenced by sunlight. The most unexpected results from our study was the discovery of highly diverse form I (*cbbL*) RubisCO gene sequences most deeply rooted in the red-like clade that either cluster with sequences from uncommon (thermo)acidophilic species [e.g., Warren Cave (I) OTU1] or possibly from completely new clades [e.g., Warren Cave (I) OTU2] (Figure 4). No form II (*cbbM*) RubisCO gene sequences were detected. Overall, these results are consistent with the caves being a fully oxic environment. The large number of diverse RubisCO sequences in the deeply rooted red clades having no or few closely related cultured representatives combined with the relatively low phylogenetic (16S SSU rRNA) diversity found in the caves (Figures 2, 3) is paradoxical and is discussed further below.

### Warren cave

Warren Cave is dark with highly oligotrophic sediments, making it an excellent example of a DOVE and a model system for studying microbial ecosystems deriving their essential energy sources from inorganic substances including those in rocks [e.g., Fe(II), Mn(II)] (Kelly et al., 2008) and volcanic gasses. That there is a low diversity of microbes in this cave makes it easier to evaluate the potential energy yielding pathways in this ecosystem from what is known about the metabolisms found in the *Chloroflexi* and *Acidobacteria* and their genomes. Mt. Erebus magmatic gas emissions are rich in CO_2_, contain CO and H_2_, but are essentially devoid of CH_4_, and H_2_S (Oppenheimer and Kyle, 2008; Moussalam et al., 2012). Thus gaseous CO as seen in other volcanic systems (King and Weber, 2007), H_2_ and reduced metals in the rocks appear to be the primary energy sources available to the microbial communities. To our knowledge CO has not been detected in the caves, although its absence could be attributed to efficient microbial utilization. Trace amounts of H_2_ and CH_4_ above air background have been detected in some caves (Fischer et al., 2013).

The phylogenetic affiliations of the major Warren Cave bacterial OTUs are consistent with the oligotrophic nature of the ecosystem. The phylogenetic diversity of Bacteria is low and there is an unusual abundance of two major phyla, the *Chloroflexi* and the *Acidobacteria*. There is no evidence of human contamination such as sequences of clones from microbes associated with the human body. Although the SSU rRNA clone library results cannot be taken to quantitatively indicate the abundance of a given taxon because of varying SSU rRNA gene copy number in different organisms, the dominance of these two phyla suggests a relatively low diversity environment and that these organisms are highly adapted to local conditions. In contrast, the large diversity of RubisCO cave sequences indicates there are a variety of organisms with autotrophic carbon fixation potential. Reconciling the SSU rRNA phylogeny with the abundance of RubisCO genes represents an enigma. As far as is known, *Acidobacteria* do not grow chemoautotrophically although they are recognized as being oligotrophic and possess predicted *cox* genes encoding CO dehydrogenase (CODH) (King and Weber, 2007; Ward et al., 2009), suggesting they may utilize CO to supplement their energy needs. Within the *Chloroflexi* at least one member (*Oscillochloris trichoides* DG6) has RubisCO (Ivanovsky et al., 1999) and this gene sequence groups with our newly-discovered, deeply rooted cave RubisCO sequences (Figure 4). Major groups within the *Chloroflexi* (like *Ktedonobacter racemifer*, to which two of the major Warren Cave OTUs, OTUs 1 and 3, are phylogenetically related; Figure 5) also possess the CODH large subunit gene *coxL* (DOE Joint Genome Institute, http://www.jgi.doe.gov/) (Weber and King, 2010; King and King, 2014). Thus, CO oxidation appears to be a common, but not universal trait of organisms in the phylum *Chloroflexi* (King and King, 2014) and may also be important to the energetics of Mt. Erebus DOVEs. Many aerobic CO oxidizers with *coxL* also possess RubisCO for CO_2_ fixation (King and Weber, 2007) although many are not able to grow with CO as the only energy source. Interestingly, *Ktedonobacter* and related species are the most dominant groups in the high altitude volcanoes in the Atacama desert (Lynch et al., 2012) and on cinder deposits of Kilauea volcano in Hawaii (Weber and King, 2010; King and King, 2014) clearly indicating the versatility of this group of organisms to be adapted to extremely oligotrophic conditions and withstand extreme environments. Although *Chloroflexi* and *Acidobacteria* are found relatively nearby in soils from the Antarctic Dry Valleys or the hydrothermally active Tramway Ridge (Mt. Erebus; Figure 1), their abundance there is much lower and the similarity of the sequences with the Warren Cave SSU rRNA sequences is low (<83%). We postulate that the *Chloroflexi* CO oxidizers and some of the other minor bacterial taxa are responsible for autotrophic CO_2_ fixation in this system.

But how do we reconcile the low 16S rRNA phylogenetic diversity of Warren Cave with the high diversity of RubisCO sequences? There are several possible reasons. First, the simplest explanation is that we undersampled the phylogenetic diversity of organisms by only analyzing 82 16S rDNA clones but have a better representation of the RubisCO from the 48 clones sequenced from each cave. This would be especially true if the two dominant groups, *Acidobacteria* and *Chloroflexi*, have a large number of copies of the small subunit (16S) ribosomal genes biasing our phylogenies toward those groups. In particular, the abundance of *Ktedonobacteria*-like sequences within the *Chloroflexi* may be overrepresented because they have a very large bacterial genome with eight copies of the 16S rRNA gene (Chang et al., 2011). In contrast, the *Acidobacteria* have only one or two copies (Lee et al., 2009). A second explanation is that the most abundant organisms by 16S rRNA gene analysis are not the organisms with the RubisCO genes (nor do they have the rTCA cycle for CO_2_ fixation which also would have been detected) and our PCR methods are selectively amplifying the RubisCO genes from the remaining low abundance autotrophic microbial populations present in the cave. Thirdly, although unlikely, it is possible that Warren Cave was successfully colonized by a relatively low diversity of microbes (at the phylum level), but, with time, their functional genes have further evolved and diverged relative to their ribosomal genes. Something similar to this has been proposed to explain the high level of sequence heterogeneity in soils from Mars Oasis, Alexander Island, Antarctica (Pearce et al., 2012). Finally, it could be that this discrepancy is simply due to the resolution at which we are analyzing sequences, grouping ribosomal sequences at higher taxa rankings than which the RubisCO sequences are grouped. A combination of these explanations is likely.

A concurrent study of the fungi based on ITS sequences in Warren Cave revealed moderate fungal diversity with all taxa belonging to the phyla *Basidiomycota* and *Ascomycota* (Connell and Staudigel, 2013). Many of those identified are known to function as saprophytes but fungi are also known to oxidize CO (Conrad and Seiler, 1980), abilities compatible with an oligotrophic lifestyle. One of the most common taxa found was a black yeast, *Aureobasidium pullans*, commonly found on rocks in polar habitats and other oligotrophic habitats (e.g., Atacama desert, Himalayas) (Onofri et al., 2007). Although diverse fungi were undoubtedly present, from the results so far we are not able to evaluate the relative abundance of Bacteria and fungi in any of the caves examined.

### Hubert's Nightmare

Hubert's Nightmare is a small cave system that was visited in 2008 and 2010. In 2012 it was inactive and frozen over. Because of the close proximity of Hubert's Nightmare to Warren Cave (less than 50 m, in the same slope area of Mt. Erebus) and the uniformity of sediment compositions of most other Mt. Erebus ice caves (Table 2), we expect Hubert's Nightmare to have the same sediment composition as Warren Cave. Thus, the main differences between the two caves are the temperature of the fumarolic vents where the sediments were collected (also creating differences in cave humidity) and the degree of weathering of the sediments sampled. Hubert's Nightmare sediments had both a lower temperature and amount of weathering (coarser grain size) than Warren Cave sediments, as well as a slightly higher pH. The lower temperature indicates a lower heat (and hence gas) flux from the vents. Therefore, when compared to Warren Cave, the overall gas chemistry in Hubert's Nightmare is likely to be closer to air because vent gasses will be subjected to more dilution by the atmosphere. This lower flux of potential gaseous energy sources combined with the higher pH may account for the differences in microbial community composition and cell numbers as compared to Warren Cave. Additionally, the cell abundance for Warren Cave (Table 3) may be inflated if the *Ktedonobacteria* have more copies of the SSU 16S ribosomal RNA gene (Chang et al., 2011). Although the major microbial groups represented in the clone libraries are not recognized for having chemolithoautotrophic metabolism, like in Warren Cave, there was a large diversity of type I RubisCO sequences present indicating the potential exists in the Hubert's Nightmare microbial community. We do not know whether some of these RubisCO sequences could belong to members of the *Verrucomicrobia*, at least one member of which is an autotrophic methanotroph that uses the CBB cycle for CO_2_ fixation (Khadem et al., 2011).

### Harry's Dream

Evidence for photosynthetic microbes was found in Harry's Dream, the only cave we studied that receives periodic light. Regardless, the TOC content and cell numbers were lower than found in Warren Cave, even though the TOC is comparably low for both sites (Table 5). In both caves we would expect a similar demand for the organic carbon from heterotrophic bacteria, so we do not believe this could explain the difference in TOC content or cell abundance. Rather, it could be due to the location of the fumaroles relative to the surrounding rock topology. In Harry's Dream the fumarole was located on a small ledge that is relatively elevated when compared with the surrounding topography. In Warren Cave the fumarole was in a depression located under a ledge where melt water and condensing steam running down lava pillows could help transport organic carbon and cells from surrounding areas and collect in the depression. Alternatively, the higher cell numbers for Warren Cave relative to Harry's Dream could simply reflect the greater abundance of organisms with high SSU rRNA gene copy number (e.g., the *Chloroflexi*).

A more curious aspect of Harry's Dream cave is the high abundance of 16S SSU gene sequences (OTUs 1 and 2) closely related to deeply branching cyanobacteria from a clade (WD272) that has never been characterized (Figure 3) coupled with the complete lack of correlated cyanobacteria-related RubisCO sequences. Green type RubisCO sequences were found in Harry's Dream but they were more closely related to RubisCOs from known chemolithoautotrophs (Figure 4). Thus WD272 (1) may possess RubisCO genes more closely related to chemolithoautotrophs, (2) they do not use the CBB cycle, or (3) they are not actually autotrophs.

The abundance of sequences within the phylum *Chloroflexi* in Harry's Dream led us to consider whether some of these sequences could be representative of phototrophic bacteria. However, all the sequences belong to classes other than the photosynthetic class (also called *Chloroflexi*). Nevertheless, because of the broad phylogenetic diversity represented within the phylum *Chloroflexi* and the known metabolic versatility within the class *Chloroflexi*, the possibility for photoautotrophy or photoheterotrophy within other taxa within the phylum in Harry's Dream is possible. In addition to the cyanobacteria, some *Acidobacteria* are known to be phototrophic (Bryant et al., 2007).

Two of the OTUs (6 and 13) from Harry's Dream phylogenetically clustered in the *Hydrogenophilales*, an order known for being thermophilic and capable of oxidizing hydrogen (Hyashi et al., 1999). The cultivated organisms from this order have an optimum temperature of ~50°C and are facultative chemolithoautotrophs. Although the temperature of the soils we collected was low compared to the optimum temperatures for the cultured isolates, it is not uncommon to find thermophilic bacteria or bacteria phylogenetically related to thermophilic bacteria in cold environments. Additionally, that these organisms are facultative chemolithoautotrophs would seem to be a good lifestyle for organisms living in ice caves where changing conditions such as gas flux and temperature occur throughout the year. This observation, plus the abundance of RubisCO genes in Harry's Dream that are related to the red or deeply rooted red type RubisCOs found in the other caves, suggests that similar chemolithoautotrophs, even if in minor abundance, exist in all three caves.

### Non-detection of archaeal sequences

Finally, it is noteworthy that we did not find any Archaeal SSU 16S rRNA gene sequences in our cave samples. Most environments, including all other Antarctic soils such as those found in the Dry Valleys, Mars Oasis, Tramway Ridge (Mt. Erebus) or a subglacial lake contain at least some Archaea sequences (Soo et al., 2009; Cary et al., 2010; Pearce et al., 2012, 2013). It is likely that Archaea are not very abundant, but whether they are truly completely absent in these caves will require analysis of environmental metagenomes.

## Conclusions

The conditions found in the DOVEs on Mt. Erebus can be compared to the oxic, extremely oligotrophic subseafloor sediments that underlie up to 48% of the world's oceans (D'Hondt et al., 2009). Dark ice caves such as Warren Cave exemplify an environment where the input of plant and other photosynthetic-derived organic carbon is minimal and thus provides an environment at the extreme low end of the spectrum for carbon concentration. Under these conditions microbes will be forced to scavenge any energy sources available, including gasses and inorganic compounds found in the rock. Thus, Warren Cave and other Mt. Erebus dark ice caves are environments that provide a unique opportunity to study the organisms, their metabolism and the adaptations that allow them to exist under such extreme oligotrophic conditions and allows microbiologists to further test the nutritional limits on life.

Understanding the metabolic processes in the oligotrophic subsurface biosphere has been limited by logistics of access and contamination potential from chemical and biological sources, particularly organic matter. Our results show that Mt. Erebus dark fumarolic ice caves may help us overcome this limitation in the study of highly oligotrophic dark ecosystems: (1) Low phylum-level Bacterial diversity confirms a commonly found adaptation to extreme environmental conditions; (2) primary production involves CO_2_ fixation via the CBB cycle using many novel and deep-rooted clades of Form I “red type” RubisCO enzymes; and (3) the energy driving CO_2_ fixation and supporting the ecosystem is likely derived from reducing volcanic rocks and gas emissions. Based on what is known about the physiology of *Chloroflexi* and *Acidobacteria*, the major phylotypes in Warren Cave, we hypothesize that CO oxidation is at least one of the energy yielding processes important in this DOVE and likely in the other ice caves as well. Although we can't completely rule out an external supply of organic matter, the remoteness of Mt. Erebus and our results indicating a low phylogenetic diversity and diverse RubisCO genes lead us to hypothesize that the caves are fundamentally supported by chemolithoautotrophy. While our study offers only a first glimpse at a very complex system, it is obvious that the DOVEs at Mt. Erebus and their previously undiscovered genetic diversity offers a welcome new perspective on important biogeochemical processes in the subsurface biosphere.

### Conflict of interest statement

The authors declare that the research was conducted in the absence of any commercial or financial relationships that could be construed as a potential conflict of interest.
